# The shocking consequences of hybrid epigenomes

**DOI:** 10.1186/s13059-016-0967-3

**Published:** 2016-05-05

**Authors:** William T. Jordan, Robert J. Schmitz

**Affiliations:** Department of Genetics, University of Georgia, 120 East Green Street, Athens, GA 30602 USA

## Abstract

The formation of spontaneous epialleles is poorly understood. A new study describes how the formation of epihybrids can lead to the appearance of novel epialleles.

## Introduction

DNA methylation is perhaps the best-known and best-studied chromatin modification. Occurring primarily at cytosine nucleotides in eukaryotic genomes, methylation is a reversible DNA modification that is linked with epialleles, which are genes that are identical in sequence but differ in methylation status and that are inherited between generations. A recent study by Rigal et al. [1] published in *Proceedings of the National Academy of Sciences U S A* reported the discovery of novel epialleles in hybrids that formed from crossing mutants that are defective in the maintenance of DNA methylation with wild-type plants, highlighting a potential mechanism by which natural epialleles arise.

## Maintenance of DNA methylation in plants

The flowering plant *Arabidopsis thaliana* is an ideal model system for studying DNA methylation because it can tolerate extensive genome-wide methylation changes. Unlike in mammals, DNA methylation in *A. thaliana* regularly occurs at three different sequence contexts: CG, CHG, and CHH (where H is either A, C, or T). In *A. thaliana,* CG methylation is maintained genome-wide by METHYLTRANSFERASE1 (MET1), whereas CHG and CHH methylation are maintained by CHROMOMETHYLTRANSFERASE3 (CMT3) and CMT2, respectively. De novo DNA methylation at any cytosine context is mostly a product of DOMAINS REARRANGED METHYLTRANSFERASE 2 (DRM2) and occurs as part of the RNA-directed DNA methylation pathway.

Mutations in MET1 in *A. thaliana* eliminate 99 % of all genome-wide CG methylation, yet these plants still produce viable offspring [2]. This characteristic has led to the creation of epigenetic recombinant inbred lines, or epiRILs. Crossing MET1-deficient individuals with wild-type plants creates progeny that contain mosaic methylomes that have no underlying DNA sequence changes, resulting in numerous epialleles [3]. Epialleles are known to cause widespread phenotypic variation in *A. thaliana* [4]. epiRILs are important in studying the long-term impacts of differential methylation, but the short-term impacts of epiallele formation remain poorly characterized.

## Spontaneous epiallele formation

Rigal et al. [5] previously reported that the lack of CG and CHG methylation in the large intron of *INCREASE IN BONSAI METHYLATION 1* (*IBM1*) results in impaired *IBM1* transcription. *IBM1* is a histone lysine demethylase that removes H3K9me2 from actively transcribed gene bodies, and protects genes from CHG methylation. Reduced expression of *IBM1* leads to aberrant CHG hypermethylation in thousands of genes [6]. In their recent study, published in *PNAS*, Rigal et al. [1] crossed individuals with a defective *MET1* gene—which leads to decreased *IBM1* intronic CG methylation and decreased expression—with Col-0 wild-type individuals [1]. Unexpectedly, the resulting offspring (termed epihybrids) exhibited further reduction in both *IBM1* intronic CHG methylation and mRNA levels at the *met1-*derived *IBM1* allele. This was not due to the effects of the *met1* mutation, as determined from several generations of self-pollination. Thus, the decrease in *IBM1* intronic CHG methylation is due either to interactions between the different parental epialleles or to large-scale differences in chromatin architecture between the two genomes. Self-pollinating *met1* x Col-0 epihybrid plants revealed a Mendelian inheritance pattern for the newly formed *IBM1* epiallele in the F_2_ progeny; one-quarter of the offspring produced low levels of full-length *IBM1* transcripts and possessed no methylation in the intron. This particular novel epiallele was verified by reversing the orientation of the cross. It was stably inherited for at least two consecutive generations, indicating that other epigenetic changes found in the epihybrids could be inherited in future generations.

These results provide evidence for an ‘epigenomic shock’, where the segregation of genomes with vastly different chromatin landscapes results in the formation of novel epialleles, which have the potential to alter the expression of protein-coding genes. Similar dynamics involving the crossing of different epigenomic states are likely to occur regularly in the wild, albeit at a smaller scale, and provide a possible mechanism for the creation of novel natural epialleles, as has been observed in crosses between some *A. thaliana* accessions [7].

## Transposable elements in epihybrids

**Fig 1:**
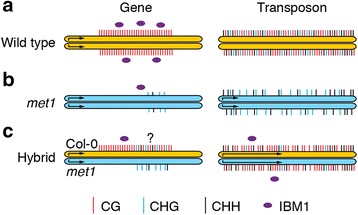
Representation of gene and transposon loci in wild-type, *met1*, and epihybrid plants. **a** Some genic regions in wild-type (Col-0) plants exhibit CG methylation (*red*) and have moderate expression levels, in part mediated by continual removal of H3K9me2 by IBM1 (*purple circles*). Transposons in wild-type plants are highly decorated with CG, CHG (*blue*), and CHH (*black*) methylation; they have no interaction with functional IBM1 and are not expressed. **b** In the *met1* mutant*,* some genic regions exhibit low levels of CHG and CHH methylation as a result of depleted IBM1, which is itself sensitive to loss of CG methylation. In *met1,* certain transposons are moderately expressed and have intermediate CHG and CHH methylation levels. **c** The Col-0-derived genic regions of epihybrid plants exhibit near wild-type levels of CG methylation on the wild-type allele (*yellow*), but might additionally contain limited CHG and CHH methylation (represented by the ‘?’). By contrast, the *met1*-derived allele does not newly acquire CG methylation. In epihybrid plants, unlike in wild-type plants, IBM1 is not entirely localized to Col-0-derived genic regions. Instead, transcriptional reactivation of certain transposons occurs in the epihybrids, which probably depletes IBM1 in genic regions and relocates it to transposons. The net effect is that these transcriptionally active transposons have reduced CHG methylation. The length of the arrow within each allele indicates transcription strength.

Rigal et al. [1] performed whole-genome bisulfite sequencing for both parents and epihybrids to compare methylation changes on a genome-wide scale. F_1_ epihybrid plants showed a substantial increase in CG methylation at certain transposons (TEs) located in pericentromeric regions (Fig. 1). A notable portion (25 %) of TEs had fully restored CG methylation in epihybrid plants, and full CG restoration was associated with re-establishment of CHG methylation. The restoration of methylation at these TEs indicates that pre-existing properties are present to facilitate this immediate return of methylation. This contrasts sharply with what occurs within genes. Roughly 100 CHG hypermethylated genes were identified in the F_1_ epihybrids that had no hypermethylation in either parent. Of these genes, 60 % are also CHG hypermethylated in *ibm1* mutants, suggesting that deficient *IBM1* activity is responsible for this aberrant CHG methylation. However, CG gene-body methylation did not deviate from expected levels (Fig. 1), indicating that regions of active remethylation are concentrated on silencing TEs in pericentromeric regions of the genome.

Most intriguingly, of the nearly 2000 re-activated TEs found in the *met1* parent (re-activated by loss of TE-silencing methylation), fewer than 3 % were transcriptionally re-silenced in the F_1_ epihybrids. This evidence indicates that the addition of CG methylation from the wild-type parent alone is not sufficient to affect the transcription levels of re-activated TEs (Fig. 1). Rigal et al. [1] propose that the poorly expressed *IBM1* allele from the *met1* parent, coupled with the re-activation and transdemethylation of transposons, recruits the remaining IBM1 to preferentially remove CHG methylation found in transcriptionally reactivated transposons in F_1_ epihybrids (Fig. 1).This titration of IBM1 away from genic regions is perhaps responsible for the observed increase in CHG gene-body methylation in F_2_ plants.

## Consequences of epigenome reprogramming

How CHG methylation is initially lost from the *met1-*derived *IBM1* allele is unknown, and will be an important area for future investigations. Other examples of widespread epigenome reprogramming have been observed in recent years when studying mutants that are deficient in some aspect of DNA methylation maintenance. In *met1* mutants, H3K9me2 has been observed in H3K27me3-targeted genes, whereas H3K9me2-depleted regions acquire H3K27me3 [8]. In another study using a mutant with a globally hypomethylated genome, known as *decrease in DNA methylation1* (*ddm1*), hypermethylation was observed at non-CG sites. Increased H3K9me2 also occurred at these sites over generational time scales, probably as the result of the misregulation of a feed-forward loop involving CMT3 and the H3K9 lysine methyltransferase KRYPTONITE [9]. Collectively, these results indicate that chromatin landscape incompatibility, as noted in *ddm1* and *met1* mutants, has the potential to alter crucial associations between histone modifications and DNA methylation, ultimately leading to the creation of altered epigenetic states.

The consequences of large-scale alterations in chromatin landscapes have also been observed in animals. The loss of H4K16ac and H4K20me3 is a hallmark of nearly every human cancer studied to date, while ectopic levels of H3K9ac and H3K4me2 can promote the formation of tumors in multiple tissue types [10]. Numerous mechanisms, including the misexpression of histone lysine methyltransferases such as the H3K9me2 methyltransferase G9a, have been found to promote the metastasis of cancerous cells [10]. By and large, the consequences of altered chromatin landscapes in promoting cancer proliferation remain largely unknown. Understanding the mechanisms and consequences of how genome integrity is disrupted is an important area of investigation that could lead to methods for engineering aberrant chromatin landscape alterations for better crop development and potentially for treating chromatin-based diseases.

## Abbreviations

CMT3: CHROMOMETHYLTRANSFERASE3
*ddm1*: *decrease in DNA methylation1*
DRM2: DOMAINS REARRANGED METHYLTRANSFERASE 2
epiRIL: epigenetic recombinant inbred line
*IBM1*: *INCREASE IN BONSAI METHYLATION 1*
MET1: METHYLTRANSFERASE1
TE: transposon
