# Erratum to: The shocking consequences of hybrid epigenomes

**DOI:** 10.1186/s13059-016-1031-z

**Published:** 2016-07-29

**Authors:** William T. Jordan, Robert J. Schmitz

**Affiliations:** Department of Genetics, University of Georgia, 120 East Green Street, Athens, GA 30602 USA

## Erratum

After the publication of this work [1] it was noticed that the wrong grant number was acknowledged in the Funding section. The correct grant number is MCB-1339194.

The author apologises for this error.
